# Draft Genome Sequence of *Lactobacillus paracasei* DmW181, a Bacterium Isolated from Wild *Drosophila*

**DOI:** 10.1128/genomeA.00545-17

**Published:** 2017-07-06

**Authors:** Austin J. Hammer, Amber Walters, Courtney Carroll, Peter D. Newell, John M. Chaston

**Affiliations:** a Department of Plant and Wildlife Sciences, Brigham Young University, Provo, Utah, USA; b Department of Biological Sciences, SUNY-Oswego, Oswego, New York, USA

## Abstract

The draft genome sequence of *Lactobacillus paracasei* DmW181, an anaerobic bacterium isolate from wild *Drosophila* flies, is reported here. Strain DmW181 possesses genes for sialic acid and mannose metabolism. The assembled genome is 3,201,429 bp, with 3,454 predicted genes.

## GENOME ANNOUNCEMENT

*Lactobacillus paracasei* is a Gram-positive anaerobic bacterium that is part of the lactic acid bacteria (LAB) group that have been studied for, among other reasons, use as probiotics (1). LAB are also commonly associated with fruit flies (2). In the present study, we sequenced and analyzed the genome of *L. paracasei* DmW181 derived from wild *Drosophila* flies.

Wild *Drosophila* flies were collected from a household kitchen in Ithaca, NY, USA (42.427481°N 76.463983°W). Flies were homogenized, and the homogenate was plated on modified de Man-Rogosa-Sharpe (MRS) medium (3). The 16S rRNA gene from a single colony was analyzed by Sanger sequencing, and a preliminary taxonomic assignment as *Lactobacillus casei* or *L. paracasei* was made. DNA was extracted using the Qiagen DNeasy blood and tissue kit (Qiagen, Hilden, Germany). Using NEBNext double-stranded DNA (dsDNA) fragmentase (NEB, Ipswich, MA, USA), DNA was fragmented and adaptors were ligated using NEBNext UltraII DNA library prep kit components, as instructed by the manufacturer (NEB). A magnetic bead size selection achieved an insert size of 1,100 nucleotides, and the library was sequenced on an Illumina HiSeq 2500 with chemistry for 250-bp paired-end reads. A total of 5,290,771 reads passed quality filtering and were used for genome assembly. Reads were assigned to one of five separate bins, each representing 200× genome coverage, and assembled into contigs using Velvet 1.2.10 (4), with k-mer lengths varying between 185 and 207. For each bin, a single assembly that maximized *N*_50_ was selected and used to assemble the final genome, as in our previous work (5). The final assembly contained 3,201,429 nucleotides in 127 contigs, with a maximum contig length of 515,598 bp and *N*_50_ of 72,562 bp. A total of 3,454 putative genes were predicted by the NCBI Prokaryotic Genome Annotation Pipeline. When we compared the shotgun genome assembly with *L. casei* and *L. paracasei* isolates available in the JSpeciesWS Web server (6), the highest similarity was 99.9% with *L. paracasei* Lpp46, so we named this strain *L. paracasei* DmW181.

Preliminary genome analysis and annotation with RAST version 2.0 (7) were performed to evaluate unique genetic features of *L. paracasei* DmW181 that could influence interactions with *Drosophila*. Comparative genomic analysis revealed genes unique to *L. paracasei* DmW181, relative to the 2 *Lactobacillus casei* isolates in RAST, *L. casei* ATCC 334 and *Lactobacillus casei* BL23. Sialic acid metabolism genes were found, including a utilization regulator and genes that participate at the interface of mannose and sialic acid metabolism. Previous research has shown that sialic acid could be important for the growth of some bacteria that colonize the mammalian gut (8–10). We suggest that future work could test if sialic acid influences colonization of the *Drosophila* gut by *Lactobacillus* or other bacteria.

### Accession number(s).

This whole-genome shotgun project has been deposited at DDBJ/ENA/GenBank under the accession no. NDXH00000000. The version described in this paper is version NDXH01000000.
